# Correlation between estimated pulse wave velocity values from two equations in healthy and under cardiovascular risk populations

**DOI:** 10.1371/journal.pone.0298405

**Published:** 2024-04-09

**Authors:** Marco Av Silva, Ana Ps De Oliveira, Ana Cs Queiroz, Amanda O. Spaziani, Leticia Ab Fernandes, Kleber A. De Oliveira, Valquiria Da S. Lopes, Manoel P. Landim, Luciana N. Cosenso-Martin, Jose F. Vilela-Martin

**Affiliations:** 1 Division of Cardiology, Department of Internal Medicine, Federal University of the Triângulo Mineiro, Uberaba, Brazil; 2 Department of Internal Medicine, São Jose do Rio Preto State Medical School, São José do Rio Preto, Brazil; 3 Department of Microbiology, Immunology and Parasitology, Federal University of the Triângulo Mineiro, Uberaba, Brazil; 4 Cardiac Diagnostic Center, Uberaba, Brazil; Universita degli Studi Magna Graecia di Catanzaro, ITALY

## Abstract

**Introduction:**

Equations can calculate pulse wave velocity (ePWV) from blood pressure values (BP) and age. The ePWV predicts cardiovascular events beyond carotid-femoral PWV. We aimed to evaluate the correlation between four different equations to calculate ePWV.

**Methods:**

The ePWV was estimated utilizing mean BP (MBP) from office BP (MBP_OBP_) or 24-hour ambulatory BP (MBP_24-hBP_). We separated the whole sample into two groups: individuals with risk factors and healthy individuals. The e-PWV was calculated as follows:

$$\begin{array}{ll} e1-PWV & =9.58748315543126\;-\;0.402467539733184*\text{age}+4.56020798207263*10^{-3}*{\text{age}}^{2}\\ & -2.6207705511664*10^{-5}{*age}^{2}*\text{MBP}+\;3.1762450559276*10^{-3}*\text{age}*\text{MBP}\\ & -1.83215068503821*10^{-2}*\text{MBP} \end{array}$$

$$e2-PWV=4.62-0.13*\text{age}+0.0018*{\text{age}}^{2}+0.0006*\text{age}*\text{MBP}+0.0284*\text{MBP}$$

We calculated the concordance correlation coefficient (Pc) between e1-PWV_OBP_ vs e2-PWV_OBP_, e1-PWV_24-hBP_ vs e2-PWV_24-hBP_, and mean values of e1-PWV_OBP_, e2-PWV_OBP_, e1-PWV_24-hBP_ and e2-PWV_24-hBP_. The multilevel regression model determined how much the ePWVs are influenced by age and MBP values.

**Results:**

We analyzed data from 1541 individuals; 1374 ones with risk factors and 167 healthy ones. The values are presented for the entire sample, for risk-factor patients and for healthy individuals respectively. The correlation between e1-PWV_OBP_ with e2-PWV_OBP_ and e1-PWV_24-hBP_ with e2-PWV_24-hBP_ was almost perfect. The Pc for e1-PWV_OBP_ vs e2-PWV_OBP_ was 0.996 (0.995–0.996), 0.996 (0.995–0.996), and 0.994 (0.992–0.995); furthermore, it was 0.994 (0.993–0.995), 0.994 (0.994–0.995), 0.987 (0.983–0.990) to the e1-PWV_24-hBP_ vs e2-PWV_24-hBP_. There were no significant differences between mean values (m/s) for e1-PWV_OBP_ vs e2-PWV_OBP_ 8.98±1.9 vs 8.97±1.8; p = 0.88, 9.14±1.8 vs 9.13±1.8; p = 0.88, and 7.57±1.3 vs 7.65±1.3; p = 0.5; mean values are also similar for e1-PWV_24-hBP_ vs e2-PWV_24-hBP_, 8.36±1.7 vs 8.46±1.6; p = 0.09, 8.50±1.7 vs 8.58±1.7; p = 0.21 and 7.26±1.3 vs 7.39±1.2; p = 0.34. The multiple linear regression showed that age, MBP, and age^2^ predicted more than 99.5% of all four e-PWV.

**Conclusion:**

Our data presents a nearly perfect correlation between the values of two equations to calculate the estimated PWV, whether utilizing office or ambulatory blood pressure.

## Introduction

Blood pressure (BP) is the most important cause of prolonged treatment and continued health care. According to data from the World Health Organization, in 2015, approximately 1.13 billion adults suffered from hypertension: with one in four men and one in five women affected [1].

Over decades, a data set has shown a positive and continued association between BP values and the occurrence of heart attacks and strokes. For every 20 mmHg increase in systolic BP and 10 mmHg increase in diastolic BP above the values of 115 mmHg and 75 mmHg, respectively, the cardiovascular (CV) risk doubles [2, 3].

Other biological markers can predict CV risks beyond BP values. Arterial stiffness has emerged as one of the most valuable, distinctive and independent predictors of cardiovascular events [4, 5].

Arterial stiffness is influenced by the material characteristics of the arterial wall. As individuals age, the elastin composition in central vessels decreases, while the recruitment of collagen fibers increases with the pressurization of vessels. Age and BP closely affect wall distensibility and, logically, arterial stiffness. As a result, arterial stiffness is lower in young adults compared to older adults and increases with higher BP values [6, 7].

In medical literature, arterial stiffness has become synonymous with pulse wave velocity (PWV). PWV is determined by dividing the distance traveled by the arterial pulse between proximal and distal vascular sites by the corresponding time. The invasive catheter measurement of PWV is only used in validation studies due to its complexity, costs and ethical limitations. Over the last two decades, several noninvasive devices have been developed and validated to measure PWV. Currently, there are some available systems that can be used for research purposes [8].

Carotid-femoral PWV (cf-PWV) represents the velocity of the pulse as it travels from the carotid and the femoral arteries. Transit times are assessed by measuring signals at the carotid and femoral arteries. This technique is still the most widely used non-invasive method and is considered the gold standard for measuring arterial stiffness. Various devices measure cf-PWW utilizing probes or tonometers to record the pulse wave in these sites. Some equipment based on this technology is now available and extensively used in published research [9, 10].

The main result of the Reference Values for Arterial Stiffness Collaboration database study was the establishment of reference values for cf-PWV. Different methodological approaches were considered in determining PWV, applying previously established conversion equations for path lengths and transit times. The correlations found between PWV and age, as well as mean BP (MBP) were highly significant in this study. Therefore, regression equations built from the database using age and MBP have shown to be able to estimate PWV [11].

Greve et al. used two equations derived from the Reference Values for Arterial Stiffness Collaboration to evaluate the predictive value of estimate PWV (ePWV). The data analyzed from two European populations indicated that an ePWV calculated from age and MBP predicted a composite cardiovascular endpoint regardless of Systematic Coronary Risk Evaluation (SCORE), Framingham risk score (FRS), and cf-PWV [12].

Recently, two other studies have provided significant findings in ePWV research. The first study also demonstrated the predictive value of ePWV for cardiovascular risk in a large Chinese population [13]. In the second study, a post hoc secondary analysis examined data from participants in the landmark Systolic Blood Pressure Intervention Trial (SPRINT). The intensive treatment was superior to standard treatment only when accompanied by a response in ePWV within the first year [14].

In those studies, researchers used the equation derived from the reference population 2010 to calculate ePWV for subjects with cardiovascular risk factors (Eq 1) and the equation derived from the healthy people in 2010 for healthy subjects (Eq 2). We hypothesized that the values of Eq 1 must be higher than those of Eq 2 because healthy individuals would typically have lower age and BP than subjects with risk factors. In fact, in a study with Marfan Syndrome of young individuals, with a mean age of 38.2 years, the values of ePWV derived from Eq 1 were significantly higher. On the other hand, in the ARIC Study, in an older cohort with a mean age of 75.2 years mean age, the ePWV from Eq 2 was substantially higher than Eq 1 [15, 16].

Therefore, from a general population, we aimed to evaluate the correlation between the two equations in middle-aged people, including treated and non-treated hypertensive individuals, as well as healthy ones.

## Methods

This study is a secondary analysis of data obtained from two cross-sectional studies conducted at a specialized center in Brazil to diagnose and treat non-communicable diseases [17, 18]. In both studies, the inclusion criteria were adults aged 18 years and above, referred to undergo ambulatory blood pressure monitoring (ABPM) due to suspected non-treated or uncontrolled hypertension following initial blood pressure measurements by a physician. The combined databases included 1541 people. For the first database, we recruited participants between 28 January and 13 December 2013, and for the second database, between 23 January 2016 and 28 June 2019. According to the pressure values measured at the specialized center and considering cutoff values of 140/90 mmHg, among the 827 individuals in the first database, 245 (29.6%) were normotensives, 181 (21.9%) were non-treated hypertensives, 197 (23.8%) were controlled hypertensives, and 204 (24.7%) were uncontrolled hypertensives. As for the 714 individuals in the second database, there were 227 (31.8%) normotensives, 233 (32.7%) non-treated hypertensives, 128 (17.9%) controlled hypertensives and 125 (17.6%) uncontrolled hypertensives. See Table 1 for the complete description of the databases.

**Table 1:** The clinical, demographic characteristics, BP, MBP and estimated PWV values in first and second databases.

	First database	Second database	
Number of participants	827	714	
Variable			P value
**Clinical characteristic**			
Age (yr)	48.3 ± 14.5	48.0 ± 14.3	0.68
White	540 (65.3)	486 (68.5)	0.18
Female sex	417 (50.4)	350 (49.1)	0.61
Healthy	81 (9.8)	86 (12)	0.16
With risk factors	746 (90.2)	628 (88)	0.16
Treated Hypertension	401 (48.5)	254 (35.6)	< 0.001
Uncontrolled Hypertension	204 (24.7)	125 (17,6)	< 0.001
Elevated BP (non-treated)	181 (21.9)	233 (32.7)	0.0031
Clinical CVD and CKD	57 (6.9)	50 (7.0)	0.93
Diabetes	75 (9.1)	76 (10.6)	0.32
Dyslipidemia	235 (28.4)	200 (28.0)	0.86
Body obesity	334 (40.4)	283 (39.7)	0.77
Smoking	76 (9.2)	51 (7.1)	0.13
Abdominal waist at risk	414 (50.1)	382 (54.4)	0.09
BMI (kg/m^2^)	29.0 ± 5.1	29.1 ± 6.5	0.73
Abdominal waist (cm)	96.6 ± 11.0	96.0 ± 12.3	0.31
**BP values**			
Systolic OBP	135 ± 16.7	135 ± 17.0	1.0
Diastolic OBP	85 ± 11.1	87 ± 11.4	< 0.001
MBP_OBP_	105 ± 12.2	106 ± 12.5	0.11
Systolic 24-hBP	125 ± 12.6	123 ± 12.3	0.001
Diastolic 24-hBP	79 ± 10.9	78 ± 10.5	0.06
MBP_24-hBP_	97 ± 10.8	96 ± 10.5	0.06
**Estimated PWV (m/s)**			
e1-PWV_OBP_	8.95 ± 1.9	8.99 ± 1.8	0.67
e2-PWV_OBP_	8.95 ± 1.8	8.98 ± 1.8	0.74
e1-PWV_24-hBP_	8.42 ± 1.7	8.30 ± 1.7	0.16
e2-PWV_24-hBP_	8.50 ± 1.7	8.40 ± 1.6	0.23

Data are demonstrated as mean ± standard deviation (SD) or absolute number (%).

BP, blood pressure; OBP, office blood pressure; 24-hBP, 24-hour ambulatory blood pressure average; MBP_OBP_, mean blood pressure of office blood pressure; MBP_24-hBP,_ mean blood pressure of twenty four hour ambulatory blood pressure average CVD, cardiovascular disease; CKD, severe chronic kidney disease; BMI, body mass index; e1-PWV, estimated pulse wave velocity from Eq 1; e2-PWV, estimated pulse wave velocity from Eq 2. The P values refer to comparisons between databases.

Prior to being fitted with an AMBP device and assisted by a trained nurse, all participants signed a written consent form to partake in the research. Later, the nurse collected demographic and clinical data, including any previous reports of clinical cardiovascular disease (CVD), acute myocardial infarction, acute coronary syndrome, coronary or other arterial revascularization, stroke, transient ischemic attack, aortic aneurysm, peripheral artery disease and severe chronic kidney disease (CKD). All subjects had their BP, weight, height, waist circumference measured and their body mass index (BMI) calculated.

Although the ePWV data from the Reference Values for Arterial Stiffness Collaboration originated from cohorts lacking established cardiovascular disease, cerebrovascular disease, or diabetes [11], we included diabetes, CVD, CKD, smokers, and obese individuals. This choice reflects a sample that more closely resembles what can be seen in everyday Brazilian physician appointments.

The study population was divided into two groups: healthy individuals and those with risk factors. Healthy individuals did not present any risk factors and a non-elevated BP (<140 and 90 mmHg). Conversely, the group with risk factors consisted of individuals with elevated BP (≥140 and-or 90 mmHg) or at least one risk factor, such as previous hypertension, dyslipidemia, diabetes, smoking, body obesity (BMI ≥ 30 kg/m2), or an increased waist circumference at risk (waist circumference > 102 cm in males and > 88 cm in females) [19].

### Blood pressure measurement and ambulatory blood pressure monitoring

During the data collection for both studies, office BP (OBP) measurements were conducted following recommended guidelines to ensure accurate pressure values [20, 21]. In the first database, a nurse performed seven consecutive BP measurements utilizing a Microlife device BP3BTOA (Onbo Electronic Co, Shenzhen, China). In the second database, a nurse assistant operated a Microlife device model BP3AC1-1PC (Onbo Electronic Co, Shenzhen, China). This device operated on Microlife Average Mode which takes three measurements in succession and calculates the average BP value. The assistant took two sets of three BP measurements sequentially.

All individuals registered twenty-four hours of ABPM using a Dyna-Mapa / Mobil-O-Graph-NG monitor (Cardios, São Paulo, Brazil), equipped with an appropriately-sized cuff on their non-dominant arm. The readings were taken every 20 minutes during the day and every 30 minutes during the night, here understood as the period between going to bed and waking up. We respected all recommended protocols strictly to ensure quality recordings [22].

### Calculation of estimated pulse wave velocity

The ePWV was calculated using the equations derived from the Reference Values for Arterial Stiffness Collaboration, incorporating age and MBP as follows [11, 12]:

$$\begin{array}{ll} e1-\text{PWV} & =9.58748315543126-0.402467539733184*\text{age}+4.56020798207263*10^{-3}*{\text{age}}^{2}\\ & -2.6207705511664*10^{-5}*{\text{age}}^{2}*\text{MBP}+3.1762450559276*10^{-3}*\text{age}*\text{MBP}\\ & -1.83215068503821*10^{-2}*\text{MBP} \end{array}$$

$$e2-PWV=\;4.62-0.13\;*\;\text{age}+0.0018\;*\;{\text{age}}^{2}+0.0006\;*\;\text{age}\;*\;\text{MBP}+0.0284\;*\;\text{MBP}$$

MBP was also calculated as diastolic BP+ 0.4*(systolic BP/diastolic BP) [11]. Thus, the values of e1-PWV and e2-PWV were obtained for the total sample, as well as separately for the groups comprising healthy individuals and those with risk factors. We used MBP from OBP (MBP_OBP_) to calculate e1-PWV_OBP_ and e2-PWV_OBP_, and MBP of twenty hours BP average (MBP_24-hBP_) to e1-PWV_24-hBP_ and e2-PWV_24-hBP._

### Statistical analysis

The databases were built using Microsoft Excel, and statistical analysis was performed using MedCalc software. The proportions, mean, and SD express the results. We utilized the D’ Agostino Pearson test to assess the normality of distribution for all continuous variables. The observed z-score shows a fairly normal distribution for all variables studied.

We utilized chi-squared statistics to compare proportions and t-tests to compare means. To investigate whether e1-PWV presents higher values of e2-PWV in the same individual group, we calculated the means of e1-PWV and e2-PWV from OBP and 24-h BP. The concordance correlation coefficient (Pc) between e1-PWV_OBP_ and e2-PWV_OBP_, as well as e1-PWV_24-hBP_ and e2-PWV_24-hBP_, was calculated for the entire sample, healthy group, and those with risk factors, seeking to explore precision (p) and accuracy (Cb) between the equations. Additionally, we used the Bland-Altman plot to analyze the agreement between all estimated PWV across the total sample, individuals classified as healthy, and those with risk factors.

Even though the Reference Values for Arterial Stiffness Collaboration highlighted that age and MBP are essential variables to determine the estimated PWV, it remains unclear in what capacity e1-PWV_OBP_, e2-PWV_OBP_, e1-PWV_24-hBP_ and e2-PWV_24-hBP_ are influenced by age and MBP values [11]. To address that issue, we employed three multilevel regression models in the entire sample. Model 1 included age and Model 2 MBP as the sole predictor variable.. In Model 3, we added MBP to the age variable. Lastly, in Model 4, we included age^2^ in addition to the variables present in Model 2 [22]. Also, we used the correlation coefficient (*r*) and scatter diagram to evaluate whether e1-PWV_OBP_, e2-PWV_OBP_, e1-PWV_24-hBP_ and e2-PWV_24-hBP_ display equal correlations with age, MBP_OBP_ and MBP_24-hBP_ within their respective ranges. The Human Research Ethics Committee of Sirio Libanes Hospital and Federal University of the Triângulo Mineiro, provided ethical approval for data collection under protocol numbers 08930813.0.0000.5461 (first database) and 61985316.9.0000.5154 (second database), respectively.

## Results

We analyzed data from 1541 participants. The first database contributed 827 participants and the second 714 participants. Table 1 provides an overview of the clinical and demographic characteristics, BP, MBP and estimated PWV values of both databases. A significantly higher prevalence of treated hypertension (48.5 vs. 35.6; p<0.001), uncontrolled hypertension (24.7 vs 17.6;; p<0.001) and higher systolic 24-hBP (123 ± 12.3 vs 125 ± 12.6 mmHg; p = 0.001) can be observed in the first database. On the other hand, elevated non-treated BP (21.9 vs 32.6; p = 0.0031) and higher diastolic BP (85 ± 11.1 vs 87 ± 11.4 mmHg;; p<0.001) are comparatively more apparent in the second database. There were no differences between the two databases in terms of the prevalence of other clinical characteristics, the proportion of healthy vs. with-risk-factors individuals, systolic OBP, MOBP, e1-PWV_OBP_ with e2-PWV_OBP_, e1-PWV_24-hBP_ with e2-PWV_24-hBP_ values. The group with risk factors consisted of 1374 (89.2%) individuals, while the ones with risk factors were 167 (10.8%).

Table 2 shows the clinical, demographic characteristics, BP, MBP and ePWV values for the entire sample, the healthy group and the group with risk factors. The participants with risk factors were found to be older than the healthy individuals (49.2±14.1–39.8±13.7 years; p <0.001). Additionally, individuals with risk factors exhibited higher abdominal circumferences (97.5±11.3–85.9±8.5 cm) p <0.001) and BMI (29.6±6.7–25.0±2.9 kg/m2); p <0.001) compared to the healthy individuals. The group with risk factors had a prevalence of treated hypertension (47.7%), uncontrolled hypertension (23.9%), elevated non-treated hypertension (30.1%), grade 2 and 3 hypertension (16.0%), clinical CVD and CKD (7.8%), diabetes (11.0%), dyslipidemia (31.7%), body obesity (44.8%), abdominal waist at risk (55.5%), and smoking (9.3%). There were no significant differences in the prevalence of female sex (50.4 vs 44.5%; p = 0.15), and white people (66.5 vs 67.1%; p = 0.87), between the group with risk factors and the healthy group.

**Table 2:** The main clinical and demographic characteristics, BP, MBP and estimated PWV values for the entire sample, the group with risk factors, and the healthy individuals.

	Whole	With risk factors	Healthy	
Number of participants	1541	1374	167	
Variable				P value
**Clinical characteristic**				
Age (yr)	48.1 ± 14.4	49.2 ± 14.1	39.8 ± 13.7	<0.001
White	1026 (66.6)	914 (66.5)	112 (67.1)	0.87
Female sex	767 (49.8)	692 (50.4)	75 (44.5)	0.15
Treated Hypertension	655 (42.5)	655 (47.7)	0 (0)	<0.001
Uncontrolled Hypertension	329 (21.4)	329 (23.9)	0 (0)	<0.001
Elevated BP (non-treated)	414 (26.8)	414 (30.1)	0 (0)	<0.001
Grade 2 and 3 Hypertension	221 (14.3)	221 (16.0)	0 (0)	<0.001
Clinical CVD and CKD	107 (7.0)	107 (7.8)	0 (0)	<0.001
Diabetes	151 (9.8)	151 (11.0)	0 (0)	<0.001
Dyslipidemia	435 (28.2)	435 (31.7)	0 (0)	<0.001
Body obesity	616 (40.0)	616 (44.8)	0 (0)	<0.001
Smoking	127 (8.2)	127 (9.3)	0 (0)	<0.001
Abdominal waist at risk	762 (49.4)	762 (55.5)	0 (0)	<0.001
BMI (kg/m^2^)	28.9 ± 5.1	29.6 ± 6.7	25.0 ± 2.9	<0.001
Abdominal waist (cm)	96.3 ± 11.6	97.5 ± 11.3	85.9 ± 8.5	<0.001
**BP values**				
Systolic OBP	135 ± 16.8	137 ± 16.8	122 ± 10.4	<0.001
Diastolic OBP	86 ± 11.3	87 ± 11.3	79 ± 7.3	<0.001
MBP_OBP_	106 ± 12.3	107 ± 12.3	96 ± 7.5	<0.001
Systolic 24-h BP	124 ± 12.5	125 ± 12.6	117 ± 9.3	<0.001
Diastolic 24-h BP	79 ± 10.7	79 ± 10.9	74 ± 7.7	<0.001
MBP_24-hBP_	97 ± 10.7	98 ± 10.8	91 ± 7.4	<0.001
**Estimated PWV (m/s)**				
e1-PWV_OBP_	8.98 ± 1.9	9.14 ± 1.8	7.57 ± 1.3	<0.001
e2-PWV_OBP_	8.97 ± 1.8	9.13 ± 1.8	7.65 ± 1.3	<0.001
e1-PWV_24-hBP_	8.36 ± 1.7	8.50 ± 1.7	7.26 ± 1.3	<0.001
e2-PWV_24-hBP_	8.46 ± 1.6	8.58 ± 1.7	7.39 ± 1.2	<0.001

Data are demonstrated as mean ± standard deviation (SD) or absolute number (%).

BP, blood pressure; Stage 2 and 3 hypertension ≥ 160 mmHg and/or ≥ 100 mmHg; OBP, office blood pressure; 24-hBP, 24-hour ambulatory blood pressure average; MBP_OBP_, mean blood pressure of office blood pressure; MBP_24-hBP,_ mean blood pressure of twenty four hour ambulatory blood pressure average; CVD, cardiovascular disease; CKD, severe chronic kidney disease; BMI, body mass index; e1-PWV, estimated pulse wave velocity from Eq 1; e2-PWV, estimated pulse wave velocity from Eq 2. The P values refer to comparisons between with risk factors and healthy groups.

The systolic OBP (137±16.8 vs 122±10.3 mmHg; p < 0.001), diastolic OBP (87±11.3 vs 79± 7.2 mmHg; p < 0.001), mean OBP (107±12.3 vs 96±7.5 mmHg; p < 0.001), systolic 24-h BP (125±12.6 vs 117±9.3 mmHg; p < 0.001), diastolic 24-h BP (79±10.9 vs 74± 7.7 mmHg; p < 0.001) and mean 24-h BP (98±10.8 vs 91±7.4 mmHg; p < 0.001), are significantly higher among those with risk factors compared to the healthy participants. Additionally, the average e1-PWV_OBP_ (9.14±1.8 vs 7.57±1.3 m/s; p <0.001), e2-PWV_OBP_ (9.13±1.8 vs 7.65±1.3 m/s; p <0.001), e1-PWV_24-hBP_ (8.50±1.7 vs 7.26±1.3 m/s; p <0.001), and e2-PWV_24-hBP_ (8.58±1.7 vs 7.39±1.2 m/s; p <0.001), were higher in those with risk factors compared to healthy individuals. There were no significant differences between e1-PWV_OBP_ with e2-PWV_OBP_ in the whole sample (8.98±1.9 vs 8.97±1.8 m/s; p = 0.88), in the group with risk factors (9.14±1.8 vs 9.13±1.8 m/s; p = 0.88) and the healthy group (7.57±1.3 vs 7.65±1.3 m/s; p = 0.57), Similarly, comparisons of e1-PWV_24-hBP_ with e2-PWV_24-hBP_ in the whole sample (8.36±1.7 vs 8.46±1.6 m/s; p = 0.09), the group with risk factors (8.50±1.7 vs 8.58±1.7 m/s; p = 0.21) and the healthy group (7.26±1.3 vs 7.39±1.2 m/s; p = 0.34) showed no significant differences. All values calculated of ePWV from MBP_OBP_ are higher than those from MBP_24-hBP_, p < 0.001.

**Fig 1:**
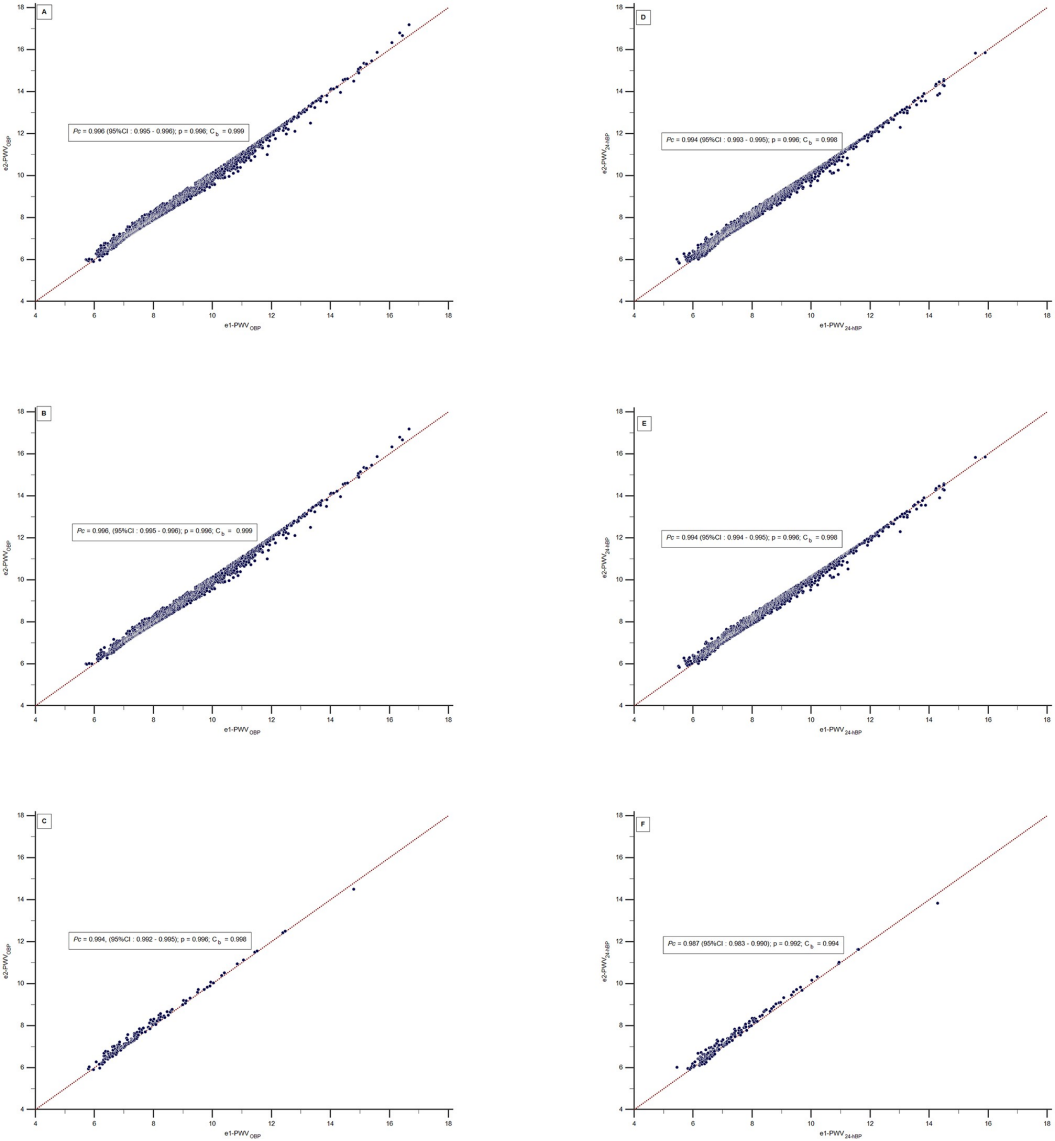
Scatter diagram depicting the concordance correlation coefficient between e1-PWV_OBP_ with e2-PWV_OBP_ and e1-PWV2_4-hBP_ with e2-PWV2_4-hBP_ values. Pc—concordance correlation coefficient; e1-PWV—estimated pulse wave velocity from Eq 1; e2-PWV—estimated pulse wave velocity from Eq 2; OBP, office blood pressure; 24-hBP, 24-hour ambulatory blood pressure average; CI—confidence interval.

The correlation between e1-PWV_OBP_ with e2-PWV_OBP_ and e1-PWV_24-hBP_ with e2-PWV_24-hBP_ was almost perfect among all participants. The *Pc* value was 0.996 (CI:0.995–0.996) equal for the entire sample and under-risk individuals and 0.994 (CI:0.992–0.995) for the healthy group. Similarly, precision and accuracy measurements presented high values. The Pearson coefficient (p) value was equally 0.996 for the three groups. The C_b_ values were 0.999 for the entire sample, with the risk factors group, and 0.998 for the healthy group (Fig 1A–1C).

Fig 1D–1F also presents the results of the correlation between e1-PWV24-hBP and e2-PWV24-hBP. In the entire sample, Pc was 0.994 (CI:0.993–0.995), p 0.996, Cb 0.998, in the under-risk group Pc was 0.994 (CI:0.994–0.995), p 0.996, Cb 0.998 and in the healthy subjects Pc was 0.987 (CI:0.983–0.990), p 0.992, Cb 0.994. The Pc values of the estimated PWV of 24-hBP were significantly lower than those from OBP in the healthy group.

**Fig 2:**
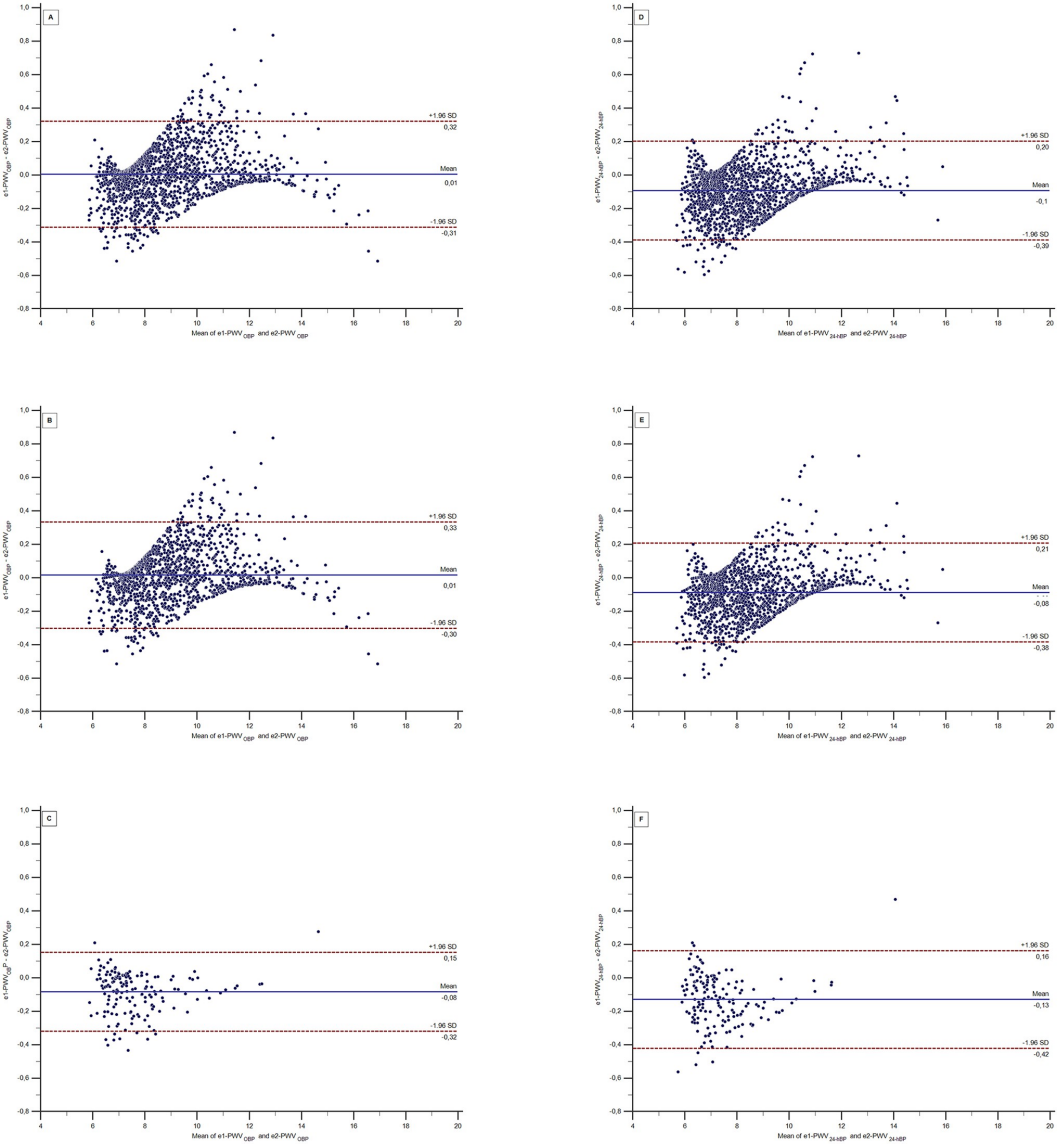
Bland Altman plot of the difference between e1-PWV_OBP_ with e2-PWV_OBP_ and e1-PWV2_4-hBP_ with e2-PWV2_4-hBP_ values. e1-PWV—estimated pulse wave velocity from Eq 1; e2-PWV—estimated pulse wave velocity from Eq 2; OBP, office blood pressure; 24-hBP, 24-hour ambulatory blood pressure average; SD—standard deviation.

Fig 2 shows the Bland-Altman plot with the mean e1-PWV and e2-PWV against the difference between e1-PWV and e2-PWV values. The mean difference between e1-PWVOBP and e2-PWVOBP was 0.01 m/s in the entire sample (A), 0.01 m/s (B) in the group with risk factors and -0.08 m/s (C) among healthy individuals. While the mean difference between e1-PWV24-hBP and e2-PWV24-hBP was -0.1 m/s in the entire sample (D), -0.08 m/s in the group with risk factors (E) and -0.13 m/s (F) among healthy individuals.

The pattern observed in all six graphs is very similar, showing fewer mean differences in low values and more differences in high values of estimated PWV. Overall, the six graphs demonstrate a good agreement between e1-PWV and e2-PWV, regardless of the group analyzed and MBP used to calculate them.

**Fig 3:**
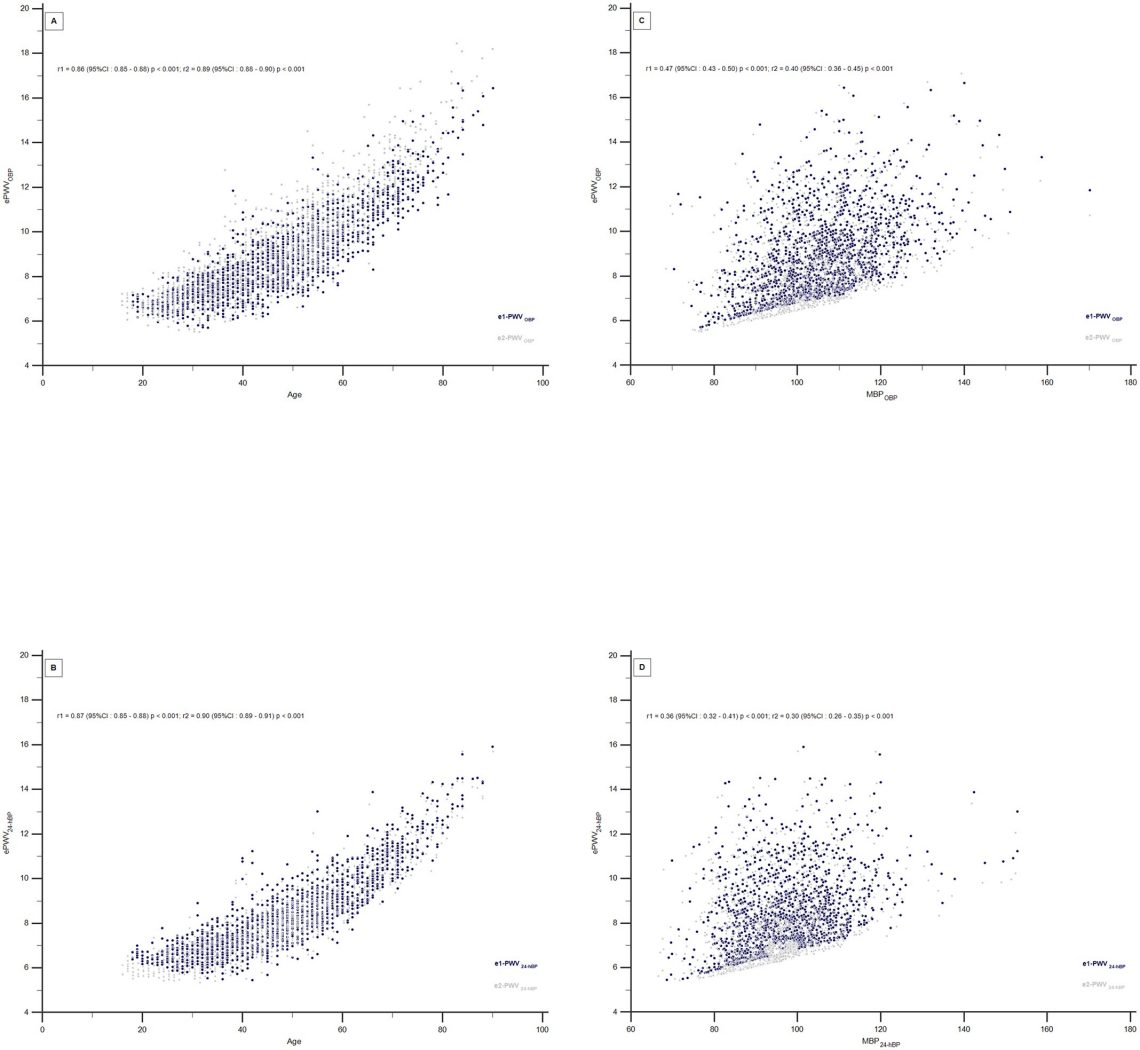
Scatter diagram exhibiting the concordance correlation coefficient from e1-PWV or e2-PWV values with age (A, B) and MBP (C, D). e1-PWV—estimated pulse wave velocity from Eq 1; e2-PWV—estimated pulse wave velocity from Eq 2; OBP, office blood pressure; 24-hBP, 24-hour ambulatory blood pressure average; MBP_OBP_, mean blood pressure of office blood pressure; MBP_24-hBP,_ mean blood pressure of twenty four hour ambulatory blood pressure average; *r*1, Pearson correlation coefficient of e1-PWV with age or MBP; *r*2, Pearson correlation coefficient of e1-PWV with age or MBP.

The scatter diagram in Fig 3 illustrates the correlation between e1-PWV_OBP_, e2-PWV_OBP_, e1-PWV_24-hBP_ and e2-PWV_24-hBP_ with age or MBP. All estimated PWV variables show stronger correlations with age than with MBP. For the corresponding values, all of them equally correlate with age, e1-PWV_OBP_ 0,86 (95%CI: 0.85–0.88), e2-PWV_OBP_ 0,89 (95%CI: 0.88–0.90), e1-PWV_24-hBP_ 0,87 (95%CI: 0.85–0.88), e2-PWV_24-hBP_ 0,90 (95%CI: 0.89–0.91). The dispersion analysis in the graph indicates similar correlations between all e1-PWV and e2-PWV across all age and MBP ranges.

Table 3 shows the results of multiple linear regression. In model 1, age explains 74.9% of e1-PWV_OBP_, 80.2% of e2-PWV_OBP_ (p = 0.09), 75.1% of e1-PWV_24-hBP_ and 85.3% of e2 PWV_24-hBP_ (p = 0.053). In the model 2, the MAP_OBP_ explains 22.2% of e1-PWV_OBP_, 16.5% of e2-PWV_OBP_ (p < 0.001) and MBP_24-hBP_ 13.2% of e1-PWV_24-hBP_ and 9.2% of e2 PWV_24-hBP_ ((p < 0.001). When we include age with MBP_OBP_ or MBP_24-hBP_ in model 3, the two variables together account for 93.6% of the e1-PWV_OBP_ and e2-PWV_OBP_ values (p = 1.0) and 90.8% of e1-PWV_24-hBP_ and 92.73% of e2 PWV_24-hBP_ (p = 0.65). Finally, in model 4, adding age^2^ the three explanatory variables predict 99.6% of e1-PWV_OBP_ and e2-PWV_OBP_ values (p = 1.0), 99.6% of e1-PWV_24-hBP_ and 99.7% of e2 PWV_24-hBP_ (p = 0.98). The R^2^ values in model 2 of e1-PWV_OBP_ and e2-PWV_OBP_ are significantly higher than those e1-PWV_24-hBP_ and e2-PWV_24-hBP_ (p < 0.001) respectively. For all others corresponding comparisons of estimated PWV, no differences in all models.

**Table 3:** Parameters of multiple linear regression models predicting values of e1-PWV_OBP_, e2-PWV_OBP_, e1-PWV_24-hBP_ and e2-PWV2_4-hBP_ from age, MBP, and age^2^.

e1-PWV_OBP_			
	R^2^	R^2^-adjusted	S_res_
**Model 1** (includes age)	0.749	0.749	0.931
**Model 2** (includes MBP_OBP_)	0.222	0.222	1.641
**Model 3** (includes age and MBP_OBP_)	0.935	0.935	0.474
**Model 4** (includes age, MBP_OBP_ and age^2^)	0.996	0.996	0.116
**e2-PWV**_**OBP**_			
**Model 1** (includes age)	0.802	0.802	0.808
**Model 2** (includes MBP_OBP_)	0.165	0.165	1.661
**Model 3** (includes age and MBP_OBP_)	0.935	0.935	0.463
**Model 4** (includes age, MBP_OBP_ and age^2^)	0.996	0.996	0.104
**e1-PWV**_**24-hBP**_			
**Model 1** (includes age)	0.751	0.751	0.850
**Model 2** (includes MBP_24-hBP_)	0.132	0.132	1.588
**Model 3** (includes age and MBP_24-hBP_)	0.908	0.908	0.517
**Model 4** (includes age, MBP_24-hBP_ and age^2^)	0.996	0.996	0.096
**e2-PWV**_**24-hBP**_			
**Model 1** (includes age)	0.813	0.813	0.716
**Model 2** (includes MBP_24-hBP_)	0.092	0.091	1.580
**Model 3** (includes age and MBP_24-hBP_)	0.927	0.927	0.448
**Model 4** (includes age, MBP_24-hBP_ and age^2^)	0.997	0.997	0.087

e1-PWV, estimated pulse wave velocity from Eq 1; e2-PWV, estimated pulse wave velocity from Eq 2; MBP_OBP_, mean blood pressure of office blood pressure; MBP_24-hBP,_ mean blood pressure of twenty four hour ambulatory blood pressure average; OBP, office blood pressure; 24-hBP, 24-hour ambulatory blood pressure average; R^2^: coefficient of determination; R^2^-adjusted: the coefficient of determination adjusted for the number of independent variables in the regression model; S_res_, residual standard deviation

## Discussion

In this study, we investigated the correlation between estimated PWV values obtained from two equations derived from a multicenter European study, aimed to determine reference values for cf-PWV using different devices. Additionally, although the equations from that study calculated ePWV from OBP, we compared values of two additional equations by replacing OBP with the 24h-BP mean in the calculations. The collaborative research showed the significance of age and BP as the primary factors influencing PWV and proposed two equations for estimating PWV, one for individuals with risk factors and another for healthy individuals [11]. Subsequent important studies demonstrated that PWV derived from these equations, regardless of BP and cf-PWV, can predict CV events and maybe serve as a therapeutic target in hypertension, beyond traditional BP measurements [12–14].

The primary finding of this study was the nearly perfect correlation observed between estimated PWV values derived from equations one and two, regardless of the MBP used in the calculation. This correlation was consistent across all groups studied, including the entire sample, individuals with risk factors and healthy individuals. There were no significant differences in the mean values of e1-PWV and e2-PWV when comparing within the same group. As anticipated, individuals in the group with risk factors were older and exhibited higher BP and MBP values than healthy individuals. Additionally, they showed significantly higher estimated PWV values, regardless of the equation used.

To clarify the primary outcome of this study, it is worth considering that no significant differences were found between e1-PWV and e2-PWV in the entire sample, as well as in the other two subgroups. The mean differences observed for e1-PWV_OBP_ and e2-PWV_OBP_ were +0.01 m/s, +0.01 m/s, and -0.08 m/s among the three groups (entire sample, with risk factors, and healthy individuals), respectively. Similar results were obtained for e1-PWV_24-hBP_ and e2-PWV2_4-hBP_ -0.1 m/s, -0.08 m/s, and -0.13 m/s. The results of this study support the notion that ePWV is strongly influenced by age and MBP values. This aligns with the regression equations derived from The Reference Values for Arterial Stiffness Collaboration, which demonstrated that PWV values increase with advancing age and higher blood pressure levels in the reference population.

As for the Reference Values for Arterial Stiffness’ Collaboration, the regression models also here employed revealed that MBP is only significantly associated with PWV through interactions with age and the square of age within the reference population. However, MBP still has a small but significant independent association with PWV in the healthy population. Based on the data obtained, it can be concluded that PWV is linearly related to BP at any age, and similarly, that it is also influenced by the quadratic term of age at any BP level [11]. Schwart et al. previously explored the impact of age and BP on PWV using the proprietary ARC Solver algorithm integrated into the Mobil-o-Graph device [23].

The link between age and MBP and cf-PWV has been demonstrated previously. Our study aimed to assess how much e-PWV is influenced by age, MBP, and age^2^. All four ePWVs show a similar dependence on age, with ePWV calculated from MBP_24-hBP_ exhibiting lesser dependency on MBP than those from MBP_OBP_. Furthermore, the multilevel linear regression analysis, showed that age, MBP and age^2^ collectively explained more than 99.6% of the variance in e1-PWV_OBP_, e2-PWV_OBP_, e1-PWV24-hBP and e2-PWV24-hBP. This suggests that these variables explain nearly 100% of the estimated PWV values derived from all equations.

The difference in ePWV between the group with risk factors and the healthy group can be easily understood. Individuals from the with-risk-factors group were, on average, 9.4 years older than the healthy group and they exhibited an average MBP_OBP_ that was 11 mmHg and MBP_24-hBP_ 7 mmHg higher. These differences were found to be quite significant.

To our knowledge, there is no published research specifically examines the association between the four equations for the ePWV in the same population. Moreover, only a few studies utilizing OBP in the calculations reported results that could compare with our data. However, equation two was utilized in a study conducted by Greeve et al. in the Danish Monica10 Cohort to assess PWV in apparently healthy individuals with cardiovascular risk factors. Surprisingly, the ePWV was associated with the measured cf-PWV, even though equation two for ePWV is recommended in healthy patients [11, 12].

Another study was designed to check whether the ARC Solver algorithm inbuilt in the Mobil-O-Graph would be able to estimate reliable PWVs in individuals with Marfan syndrome. In this study, both equations for estimating PWV were employed and the average values obtained from them did not demonstrate any significant difference [15]. Taken together, the data from these studies help us to explain and reinforce the results obtained in our study which presents both strengths and limitations.

One of the main strengths of our study is the high quality of BP and ABPM measurements, as accurate MBP is essential to determine estimated PWV. The technique used for BP measurements strictly followed the recommended guidelines for ensuring reliable measurements. The BP measurement protocol employed in both databases was consistent and aligned with protocols used in previous studies that have demonstrated an association between e-PWV and CV risk [12–14]. Thus being, we believe the ePWV values obtained in our study to be precise and accurate. Additionally, ours is the first study to use MBP derived from 24h-BP to calculate PWV based on the equations derived from the Reference Values for Arterial Stiffness’ Collaboration. Nevertheless, the main limitation of our work is its cross-sectional design and the fact that it involved a secondary analysis of data. The study population primarily consisted of individuals with treated hypertension as well as undiagnosed individuals with untreated hypertension, owing to the fact that said individuals had been referred to a specialized center for ABPM. As a consequence, the generalizability of our findings to a primary care population may be limited, and the results should be interpreted with caution in that context.

However, it is important to note that cf-PWV, despite being the gold standard method for measuring arterial stiffness, devices used to measure it, such as the Complior system or SphygmoCor system, are expensive and the cf-PWV measurements require adhering to standardized protocols. These factors, along with the need for trained personnel and the time involved in conducting the procedure, make cf-PWV measurements less feasible in general practice [24]. The ePWV, on the other hand, can be calculated using only age and MBP, and hence offers a potential alternative to cf-PWV. The estimated PWV is more affordable and easier to use in healthcare practices. The results of our study provide valuable insights for research and clinical practice by simplifying the calculation of the estimated PWV. By using a single equation, the estimated PWV can be more readily applied in healthcare settings.

Finally, the calculation of ePWV from values of 24h-BP presents future perspectives in the research field. Future research could help determine whether the ePWV from MBP_24h-BP_ would enhance the prediction of cardiovascular events of MBP_OBP_ and correlate better with cf-PWV as well other target organ injuries such as ventricular hypertrophy, carotid plaques, intima-media thickening, renal function and microalbuminuria.

## Conclusion

In summary, whether utilizing office or ambulatory blood pressure to calculate estimated pulse wave velocity, our study revealed an almost perfect correlation between values obtained from two independent equations for estimated PWV. These findings bear significant implications for the routine utilization of ePWV calculation in healthcare.

## Supporting information

S1 File (XLSX)
